# Depletion of Regulatory T Cells Augments a Vaccine-Induced T Effector Cell Response against the Liver-Stage of Malaria but Fails to Increase Memory

**DOI:** 10.1371/journal.pone.0104627

**Published:** 2014-08-12

**Authors:** Maria del Rosario Espinoza Mora, Christiane Steeg, Susanne Tartz, Volker Heussler, Tim Sparwasser, Andreas Link, Bernhard Fleischer, Thomas Jacobs

**Affiliations:** 1 Bernhard Nocht Institut für Tropenmedizin, Abteilung Immunologie, Hamburg, Germany; 2 Universitätsklinikum des Saarlandes, Klinik für Innere Medizin III, Homburg, Germany; 3 TWINCORE, Zentrum für Experimentelle und Klinische Infektionsforschung, Institut für Infektionsimmunologie, Hannover, Germany; Federal University of São Paulo, Brazil

## Abstract

Regulatory T cells (T_reg_) have been shown to restrict vaccine-induced T cell responses in different experimental models. In these studies CD4^+^CD25^+^ T_reg_ were depleted using monoclonal antibodies against CD25, which might also interfere with CD25 on non-regulatory T cell populations and would have no effect on Foxp3^+^CD25^−^ T_reg_. To obtain more insights in the specific function of T_reg_ during vaccination we used mice that are transgenic for a bacterial artificial chromosome expressing a diphtheria toxin (DT) receptor-eGFP fusion protein under the control of the foxp3 gene locus (depletion of regulatory T cell mice; DEREG). As an experimental vaccine-carrier recombinant *Bordetella* adenylate cyclase toxoid fused with a MHC-class I-restricted epitope of the circumsporozoite protein (ACT-CSP) of *Plasmodium berghei (Pb*) was used. ACT-CSP was shown by us previously to introduce the CD8^+^ epitope of *Pb*-CSP into the MHC class I presentation pathway of professional antigen-presenting cells (APC). Using this system we demonstrate here that the number of CSP-specific T cells increases when T_reg_ are depleted during prime but also during boost immunization. Importantly, despite this increase of T effector cells no difference in the number of antigen-specific memory cells was observed.

## Introduction

The liver stage of malaria is an attractive target for vaccination. During the liver stage sporozoites invade hepatocytes where they replicate without provoking clinical illness. After the infected hepatocytes burst, merozoites are released into the blood stream and infect red blood cells, where they undergo a massive replication leading to the symptoms of malaria. Infected hepatocytes present plasmodial antigens on MHC class I molecules where they can be recognized by antigen-specific CD8^+^ T cells [1], [2]. A high level of protection was induced using irradiated sporozoites [3] IFNγ production by these cells is implicated in protection [4], [5]. More recently genetically modified sporozoites were used as vaccines which induce antigen-specific T cells but are not capable of establishing the infectious cycle [6], [7]. *P. berghei* is a worldwide employed infection model for malaria in mice. The efficacy of experimental vaccines can be tested employing infection of BALB/c mice with *P. berghei* sporozoites [8]. Here a peptide of the circumsporozoite protein (CSP) was identified that is presented on H-2K^d^. Thus in this model the number and function of antigen-specific T cells [9] can be monitored. Up to now several different methods were used to induce CSP-specific T cells [10]. Some of these strategies indeed induce promising numbers of CSP-specific CD8^+^ T cells but the degree of protection often varies. Up to now the most promising strategies rely on heterologous prime/boost immunization [11].

The adenylate cyclase toxoid (ACT) of *Bordetella pertussis* is capable of delivering its catalytic domain and inserted cargo CD8^+^ T cell epitopes into the cytosol of CD11b-expressing professional antigen-presenting cells. Thus recombinant and detoxified ACT containing different epitopes was repeatedly used for delivery into the MHC class I presentation pathway to generate CD8^+^ T cells against model antigens [12], which demonstrates the versatility of this tool as antigen-delivery system. We used recombinant detoxified ACT containing an epitope of *Plasmodium berghei* CSP (ACT-CSP) in other studies to induce high numbers of IFNγ secreting CD8^+^ T cells, which confer sterile immunity against sporozoite challenge when combined with a blockade of CTLA-4 or using a heterologous prime/boost approach with CSP-espressing *Salmonella* [13].

Regulatory T cells (T_reg_) were shown to inhibit immune responses and thereby play a central role in peripheral tolerance. T_reg_ also attenuate the immune response during infections and vaccination. In naïve mice CD4^+^Foxp3^+^ T cells express CD25. Thus antibodies against CD25 have been used to deplete CD4^+^CD25^+^ T_reg_ cells [14], [15]. Recent studies have shown that a depletion of CD4^+^CD25^+^ T_reg_ by antibodies led to an increased number of vaccine-induced T cells [16]. Understanding the exact role of T_reg_ during vaccination is hampered due to the multiple effects anti-CD25 might have also on non-regulatory T cells that express CD25. Until recently no tools were available to selectively deplete T_reg_. Thus we employed transgenic mice in which Foxp3^+^ T_reg_ can be specifically depleted to study the specific function of T_reg_ during vaccination against the liver-stage of *P. berghei* [17]. These mice are transgenic for a bacterial artificial chromosome expressing a diphtheria toxin (DT) receptor-eGFP fusion protein under the control of the foxp3 gene locus. This allows a selective depletion of Foxp3^+^ T_reg_ by DT injection.

Collectively our data demonstrate that CD4^+^Foxp3^+^ T_reg_ dampen the magnitude of a CSP-specific CD8^+^ T cell response that was induced by vaccination during priming and/or boosting. In addition, we demonstrated that an enhanced number of T effector cells are not necessarily accompanied by an increased number of memory T cells.

## Materials and Methods

### Mice and parasites

Male DEREG-mice [17] were crossed with female Balb/c mice in the animal facility of the Bernhard Nocht Institute for Tropical Medicine. The resulting offsprings were typed by PCR and flow cytometry. In each experiment hemizygotic DEREG mice were used with sex and age-matched wildtype littermates as controls. For some experiments we used the F1 generation of male DEREG mice on BALB/c background and female C57BL/6 wild type mice (H-2Kb). The resulting F1 generation expresses H-2Kd and H-2Kb, which allows the simultaneous analysis of T cell responses against the malaria CSP-epitope (SYIPSAEKI corresponding to CSP 245–253) and the SIINFEKL epitope of ovalbumin. Animal maintenance and experimental procedures were all approved by the the relevant regulatory agency in Germany, the “Amt fuer Gesundheit und Verbraucherschutz of the Hansestadt Hamburg”. The procedures followed were in accordance with the ethical standards of the review board of the Bernhard-Nocht-Institute for Tropical Medicine and the Ethic Comission of the state of Hamburg as well as the related recommendations of the EU.

For all experiments 6–8 weeks old mice were used. *Plasmodium berghei* ANKA was maintained by alternating cyclic passage of the parasite in *Anopheles stephensi* mosquitoes and BALB/c mice at the mosquito colony of the Bernhard Nocht Institute for Tropical Medicine. Sporozoites were collected by manual dissection of infected mosquito salivary glands in minimal essential medium (MEM) 18–21 days after the mosquito had taken an infectious blood meal.

### Depletion of Treg cells

For depletion of T_reg_ cells, DEREG and control mice were injected i.p. with 1 µg diphtheria toxin (Merck) diluted in endotoxin-free PBS for three consecutive days, starting on day 1 after prime or boost immunization.

### ACT-CSP toxoid construction and purification

The construction of ACT-CSP was described in a previous study [9]. The amino acid sequence VRVRKNNDDSYIP SAEKILEFVKQ, which comprises the MHC I epitope SYIPSAEKI corresponding to CSP 245–253, was inserted at position 336 into the catalytic domain of the adenylate cyclase of *B. pertussis*. The resulting ACT-CSP also includes the OVA-derived SIINFEKL epitope, presented in the context of H-2K^b^ (corresponding to residues 257 to 264 from OVA). The detoxified ACT-CSP was produced in the *E. coli* strain XL1-Blue (Stratagene) transformed with the appropriate plasmid construct.

### Immunization and challenge

Mice were immunized i.p. with a single dose of 20 µg ACT-CSP diluted in 200 µl of phosphate buffered saline (PBS) on day 0. Boost immunization was performed 14 days after prime immunization. Challenge was performed i.v. seven days after prime or boost immunization using 1000 *Pb* sporozoites. For experiments concerning induction of memory responses the challenge was performed at later time points as indicated. Mice were examined every day and parasitemia was determined every two days by light microscopy of blood smears with Wright’s stain (Sigma, Taufkirchen, Germany).

### Quantification of liver-stage burden

Quantification of liver-stage parasite burden was performed as described previously (Mol. Biochem. Parasitol. 2001, 118, p233–245). Briefly, at 30 h post-challenge, livers were perfused with PBS and removed. Total RNA was extracted with Trizol (Invitrogen, Darmstadt, Germany) according to the manufacturers instructions. RNA was transcribed using random hexamer primer and RevertAid H minus reverse transcriptase (Thermo Scientific, St. Leon-Rot, Germany) according to the manufacturers instructions. The resulting cDNA was amplified using the following primers: 5′-GGATGTATTCGCTTTATTTAATGCTT-3′ and 5′-CACGCGTGCAGCCTAGTAT-3′ for the detection of 18S rRNA of PbA. As reference gene mouse GAPDH was amplified with the primers 5′- GGGTGTGAACCACGAGAAAT-3′ and 5′-CCTTCCACAATGCCAAAGTT-3′. Cycling conditions were as following: 15 min 95°C, 40 cycles at 95°C for 15 s, 50°C for 20 s and 68°C for 20 s. For each cycle a melting curve analysis was performed with a ramp from 67° to 95°C. The relative concentration of *P. berghei* 18S rRNA was determined with the comparative C_t_ method (delta delta C_t_).

### Isolation of splenocytes and PBL

Spleens were removed at the indicated time points and RBCs were lysed by addition of ammonium chloride. Blood was collected from the tail vein and RBCs were lysed by addition of ammonium chloride.

### IFNγ-ELISPOT

Single cell suspensions (2×10^5^/well) were cultivated in Millipore HTS HA plates coated with anti-mouse IFNγ. Cells were stimulated with 1 µg/ml CSP_245–253_ peptide and in some experiments with 0.1 µg OVA 257–264. Supernatants were removed after 18 hours and the number of IFNγ-producing cells was determined using ELISPOT, as described elsewhere [18]. Antibodies and Avidin-HRP were purchased from BD Pharmingen (Heidelberg, Germany). The synthetic peptides SYIPSAEKI and SIINFEKL, corresponding to CD8+ T-cell epitopes CSP 245–253 and OVA 257–264 respectively, were obtained from MWG (Ebersberg, Germany).

### Analysis of cytokine production and proliferation

Single-cell suspensions (10^5^ cells/well) were cultivated in 96-well plates. Cells were stimulated with 0.01 µg/ml SIINFEKL, 0.1 µg/ml CSP, 3 µg/ml anti-CD3 or medium RPMI as control. After 48 h, supernatants were removed and the IFNγ concentration was determined by indirect sandwich enzyme linked immunosorbent assay (ELISA). Antibodies and cytokine standard were purchased from R&D Systems (Wiesbaden, Germany). Proliferation was analyzed after 24 h by incorporation of [^3^H] thymidine. Therefore, cells were pulsed with 0.5 mCi of [^3^H] thymidine for 6 h.

### Antibodies and flow cytometry

The following antibodies were purchased from BD Pharmingen (Heidelberg, Germany): APC-, FITC- or PE-labeled rat anti-mouse IFNγ, IL-10, CD4, CD8a, CD25, CD62L, and FITC-labeled hamster anti-mouse CD69. APC-labeled rat anti-mouse TNFα, PE-labeled rat anti-mouse IL-2, PE- and FITC-labeled anti-mouse Foxp3, as well as the appropriate isotype controls were from eBioscience (San Diego, CA). The frequency of CSP-specific T cells was determined using an APC-labeled H2-Kd pentamer loaded with the CSP 245–253 peptide (Proimmune, Oxford, UK). Intracellular Foxp3 staining was performed with the Foxp3 staining set (eBioscience, San Diego CA), according to the manufacturer’s instructions. Dead cells were excluded by propidium iodide staining. Data were acquired on a FACSCalibur flow cytometer (Becton Dickinson, Mountain View, CA) and analyzed with the CellQuest program (Becton Dickinson). Facs plots are minimally adjusted in brightness and contrast using Adobe Photoshop CS6 (Adobe Systems, San Jose, CA).

### Statistical analysis

Statistical analysis was done with GraphPad PRISM (GraphPad Software Inc., San Diego, USA). The Student’s t-test was used for measuring the significance of difference between two distributions. statistical significance. Multiple groups were compared using a one-way or two-way analysis of variance (ANOVA). The Mann-Whitney test and Bonferroni Multiple Comparison test were used as a post hoc comparison of the ANOVA. For survival analysis we employed Kaplan-Meier estimator function for lifetime data. If not otherwise stated, data are expressed as means ± SD. Statistical significance was indicated as followed: ****p<0.0001; ***p<0.001; **p<0.01; *p<0.05.

## Results

### Kinetics of T_reg_ depletion in DEREG mice

**Figure 1:**
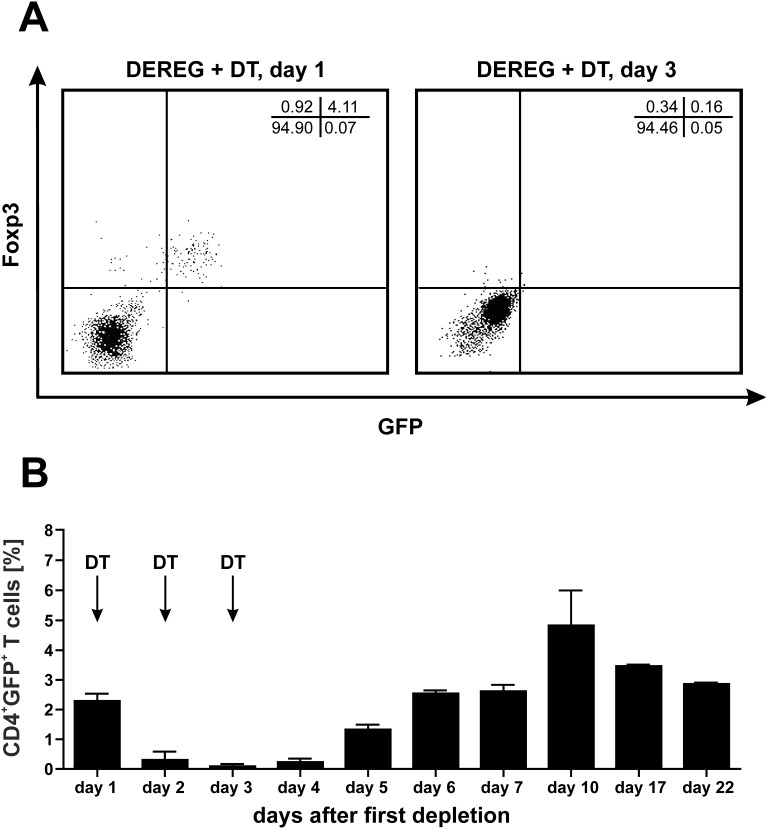
Kinetics of T_reg_ depletion in DEREG mice. In DEREG mice on BALB/c background Foxp3^+^ T_reg_ can be indentified by their eGFP expression using flow cytometry. The consecutive application of DT i.p. over three days leads to a depletion of eGFP^+^Foxp3^+^ T_reg_ within 72****h (A). For immunization experiments we used an optimized protocol where mice were treated with DT on days +1, +2 and +3. The percentage of CD4^+^GFP^+^ T_reg_ in the blood was measured in two F1 DEREG C57BL/6 x BALB/c mice by flow cytometry at the indicated time points (B). Data is expressed as mean +**/−** SEM in relation to all cells in the lymphocyte gate as defined by the respective forward- and sideward scatter.

GFP^+^Foxp3^+^ T_reg_ can be rapidly depleted in DEREG mice by i.p. injection of DT. The percentage of CD4^+^Foxp3^+^ T_reg_ cells dropped from approximately 3% to under 1.5% in blood and spleen when analyzed 12 h after a single i.p. application of DT (data not shown). We tested several protocols for the depletion of T_reg_. Most efficient was a treatment on three consecutive days [18]. Using this protocol the T_reg_ numbers were significantly reduced for almost 5 days. Thus we decided to define a protocol were we inject DT on days +1, +2, +3 (Fig. 1 A, Fig. S1). Using this schedule T_reg_ were strongly reduced from day +1 to +5 when the strongest expansion of vaccine-induced T cells could be expected (Fig. 1 B). Modified schedules i.e. starting from –1 and/or prolonged depletion do not lead to statistical significant enhanced T cell responses (data not shown). The number of eGFP^+^/Foxp3^+^ T cells started to increase from days +5 to +6 (Fig. 1 B). On later time points depleted mice have similar numbers of eGFP^+^/Foxp3^+^ T_reg_ compared to DT treated non transgenic littermates control mice.

### Functional analysis of reappearing T_reg_

**Figure 2:**
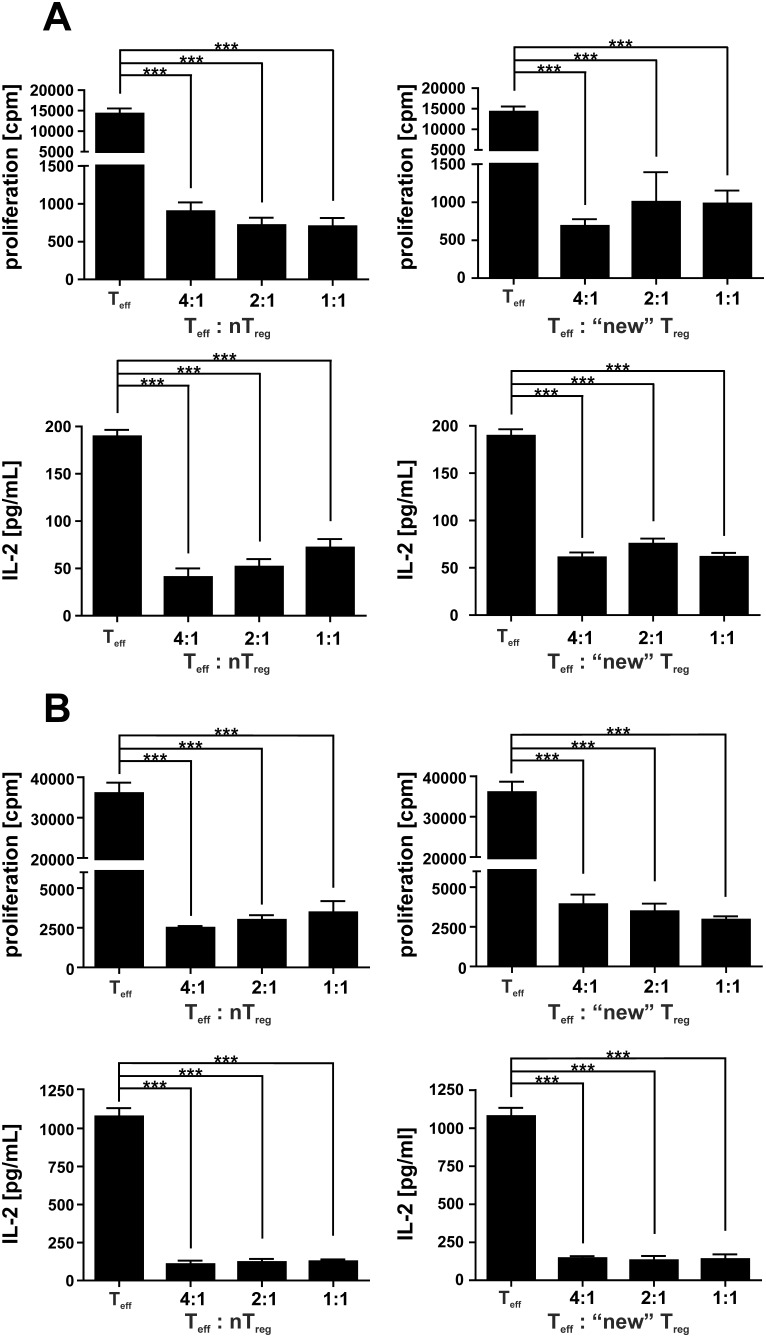
T_reg_ that repopulated depleted DEREG-mice are functional suppressors. (A) A F1 DEREG C57BL/6 x BALB/c-mouse were depleted by three consecutive DT injections and 30 days after depletion the T_reg_ that repopulated the animals were sorted by flow cytometry due to their eGFP-expression (“new”T_reg_). Similarly GFP^+^T_reg_ were sorted from an untreated F1 DEREG mice. Purity of both T_reg_ preparation was>95%. Naïve spleen cells were stimulated with anti-CD3 and incubated with different amounts of the initial GFP^+^T_reg_ (natural T_reg_, nT_reg_) before depletion and the GFP^+^ “new” T_reg_ that reappear after depletion. After 48****h the supernatants were harvested and IL-2 production was measured by ELISA. The proliferation of cells was measured by ^3^H–Thymidin incorporation. Data are expressed as mean +**/−** SD. The experiment was repeated twice with similar results. (B) A similar assay was performed using either the initial T_reg_ purified from F1 DEREG C57BL/6×BALB/c mice or those “new” T_reg_ that reappear in these mice after depletion. Initial T_reg_ or “new” T_reg_ were incubated with different amounts of spleen cells from OT-1 T cell receptor transgenic mice, which were subsequently stimulated with SIINFEKL-peptide. After 48 h IL-2 production and proliferation of cells was measured. Data are expressed as mean +**/−** SD. The experiment was repeated once with similar results. ***p<0.0001.

After transient depletion of T_reg_ by DT injection GFP^+^Foxp3^+^ T_reg_ cells repopulate depleted mice rapidly (Fig. 1B). Currently it is not known if this is due to proliferation of remaining T_reg_, *de novo* repopulation by thymic derived T_reg_ or due to conversion of conventional T cells. However, we were interested if T_reg_ that repopulate depleted DEREG mice (“new” T_reg_) have the same regulatory capacity as the initial T_reg_ population. To this end BALB/c DEREG mice were depleted by three consecutive DT injections and 30 days after depletion the T_reg_ that repopulated the animals were sorted by flow cytometry due to their eGFP-expression. Similarly GFP^+^ T_reg_ were sorted from untreated DEREG mice. Spleen cells of untreated mice were stimulated with anti-CD3 and incubated with different amounts of the initial GFP^+^ T_reg_ before depletion and the “new” T_reg_ that reappear after depletion. Both types of T_reg_ exhibited similar suppressive capacity and inhibited IL-2 production and proliferation (Fig. 2 A). Similar assays were performed using either the initial T_reg_ purified from F1 DEREG C57BL/6×BALB/c mice or those that reappear in these mice after depletion. Initial T_reg_ or “new” T_reg_ were incubated with different amounts of spleen cells from OT-1 T cell receptor transgenic mice, which were subsequently stimulated with SIINFEKL-peptide. Again, both types of T_reg_ exhibit a strong suppressive capacity (Fig. 2 B). Thus T_reg_ that repopulate mice after transient depletion were functional.

### Effects of T_reg_ depletion during prime immunization

**Figure 3:**
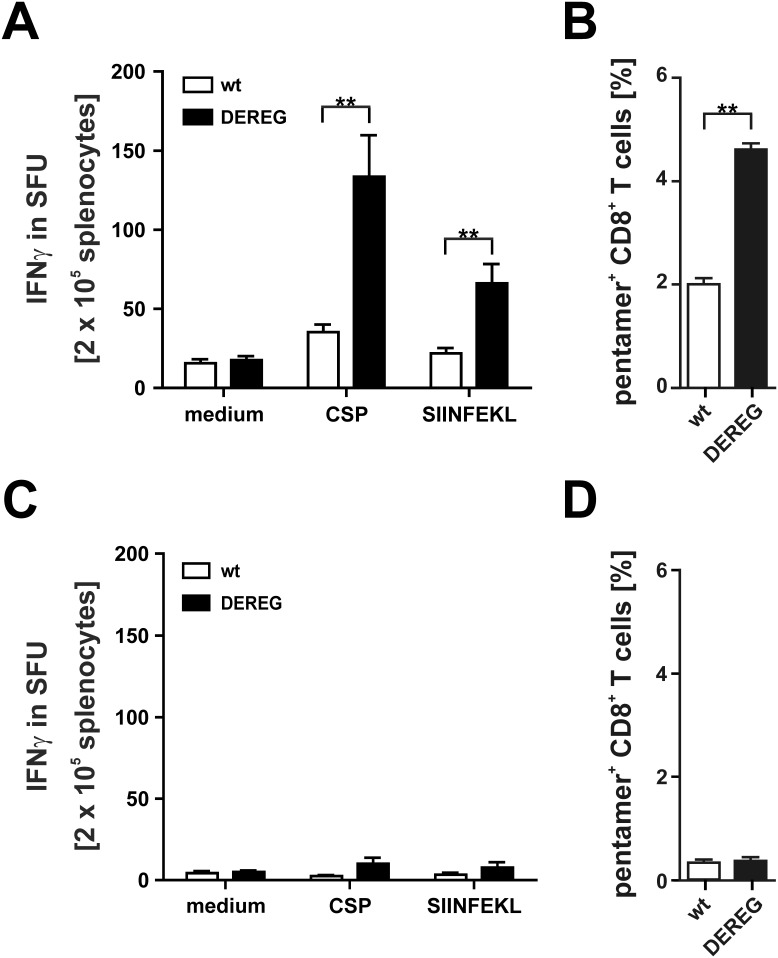
Effect of T_reg_ depletion on the efficacy of a primary immunization. (A) To analyze the effect of T_reg_ depletion on the efficacy of immunization, we immunized the F1 generation from male DEREG-mice on C57BL/6 background and female wildtype BALB/c mice (F1 DEREG) on day 0 using i.p. application of 20** µ**g of an adenylate cyclase toxoid (ACT-CSP) that carries an insert of the *P. berghei* CSP-derived CD8^+^ T cell epitope SYIPSAEKI presented on H-2K^d^ (corresponding to residues 245 to 253 from CSP) and the OVA-derived SIINFEKL epitope, presented in the context of H-2K^b^ (corresponding to residues 257 to 264 from OVA). On day +1, +2, and +3 immunized F1 DEREG and F1 non-transgenic littermates were treated with DT. On day +7, spleen cells were restimulated with CSP-peptide or OVA-peptide and IFNγ production in spot forming units (SFU) was analyzed using ELISPOT. Data are expressed as mean +**/−** SD of 5 mice. This experiment was repeated twice with similar results. (B) Percentage of CD8^+^CSP^+^ spleen cells stained using PE-labeled anti-CD8 and APC-labeled MHC-I-pentamer loaded with CSP-peptide to detect CSP-specific CD8^+^ T cells. The mean of five immunized F1 DEREG mouse or a F1 non-transgenic littermates is shown. The percentage of CD8^+^CSP^+^ T cells is expressed as mean +**/−** SD of whole lymphocytes. (C) A similar experiment was performed using again F1 DEREG mice and F1 non-transgenic littermates that were treated on days +1, +2, and +3 with DT but without previous immunization. Data are expressed as mean +**/−** SD of 5 mice. This experiment was repeated once with similar results. (D) Percentage of CD8^+^CSP^+^ spleen cells stained using PE-labeled anti-CD8 and APC-labeled MHC-I-pentamer loaded with CSP-peptide to detect CSP-specific CD8^+^ T cells. The mean of five immunized F1 DEREG mouse or a F1 non-transgenic littermates is shown. The percentage of CD8^+^CSP^+^ T cells is expressed as mean +**/−** SD of whole lymphocytes. **, p<0.01.

To analyze the effect of T_reg_ depletion on the efficacy of immunization, we used an adenylate cyclase toxoid (ACT-CSP) that carries an insert of the *P. berghei* CSP-derived CD8^+^ T cell epitope SYIPSAEKI presented on H-2K^d^ (corresponding to residues 245 to 253 from CSP) and in addition the OVA-derived SIINFEKL epitope, presented in the context of H-2K^b^ (corresponding to residues 257 to 264 from OVA) [9], [13]. For initial immunization experiments we used the F1 generation from male DEREG-mice on C57BL/6 background and female wildtype BALB/c mice (F1 DEREG). Cells from these mice express the MHC class I molecule H-2K^b^ and H-2K^d^. This allows the simultaneous characterization of T cell responses against *P. berghei*-CSP (H-2K^d^) and the SIINFEKL (H-2K^b^) peptide used as model antigen in various studies. We have previously shown that a single application of ACT-CSP can induce IFNγ producing CSP-specific CD8^+^ T cells. However, the number of CSP-specific CD8^+^ T cells remained under a threshold needed for protection unless it was combined with a blockade of CTLA-4 [9]. Accordingly, when F1 wildtype mice were immunized i.p. with ACT-CSP and treated with DT, an induction of CSP- and SIINFEKL-specific T cells in spleens was found to produce IFNγ upon restimulation with the respective peptide. However, when F1 DEREG littermates were immunized and treated with DT a strong increase in IFNγ production was observed (Fig. 3 A). To further evaluate if the number of CSP-specific CD8^+^ T cells also increased, we specifically stained these cells using the MHC-class I multimer technology and analyzed the CSP-specific T cells subsequently by flow cytometry. Here we also found that upon depletion of T_reg_ an increased number of CSP-specific T cells after immunization with ACT-CSP was induced (Fig. 3 B). Not immunized control mice shown no increased specific response upon restimulation. (Fig. 3 C and D). These data show that a transient depletion of T_reg_ augments the priming of antigen-specific CD8^+^ T cells.

**Figure 4:**
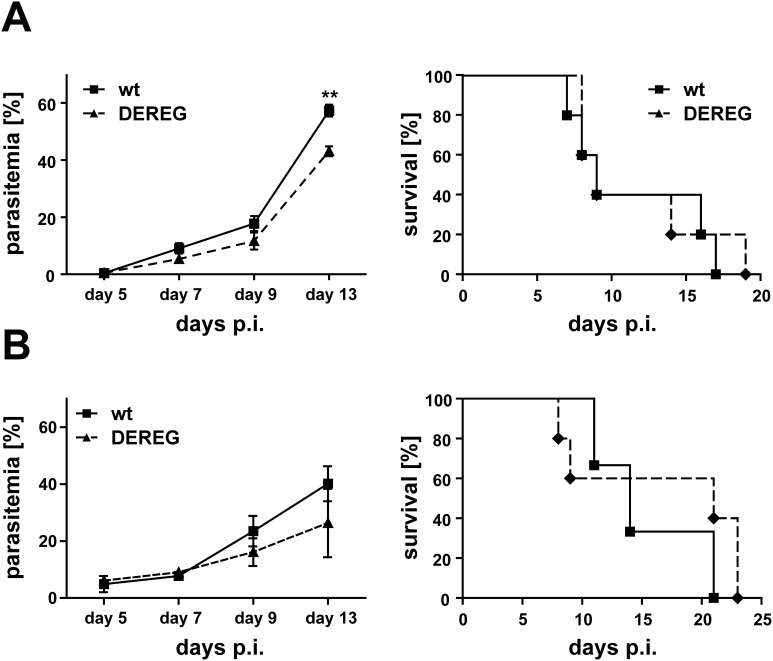
T_reg_-depletion during primary immunization confers only a partial protection against challenge with *P. berghei* sporozoites. (A) To analyze the effect of T_reg_ depletion on protection DEREG-mice were immunized on day 0 using i.p. application of 20** µ**g ACT-CSP. On day +1, +2, and +3 immunized DEREG-mice and non-transgenic littermates were treated with DT. At day 9 post immunization both groups of mice were challenged with 1000 *P. berghei* sporozoites i.v. The resulting parasitemia was monitored at the indicated time points. Parasitemia is expressed as mean +**/−** SD of five mice per group. This experiment was repeated twice with similar results. A single application of ACT-CSP to DT-treated wild type mice delayed the onset of the blood-stage. (B) The transient depletion of T_reg_ in DEREG mice without immunization did not influence the parasitemia. **, p<0.01.

The observation that a single immunization with ACT-CSP in combination with the depletion of T_reg_ already induces a high number of CSP-specific CD8^+^ T cells, prompted us to consider whether this regimen would be capable to induce protection. To this end BALB/c DEREG mice and their non-transgenic littermates were immunized with ACT-CSP and treated as described with DT to deplete T_reg_. At day 7 post immunization both groups of mice were challenged with *P. berghei* sporozoites (Fig. 4). The course of the infection was analyzed at the indicated time points. In the DEREG group, a lower increase in the levels of parasitemia after challenging was observed (Fig. 4 A). No significant differences in the survival time of the two groups could be observed. It is noteworthy that a transient depletion of T_reg_ in DEREG mice without immunization did not influence the parasitemia (Fig. 4 B).

**Figure 5:**
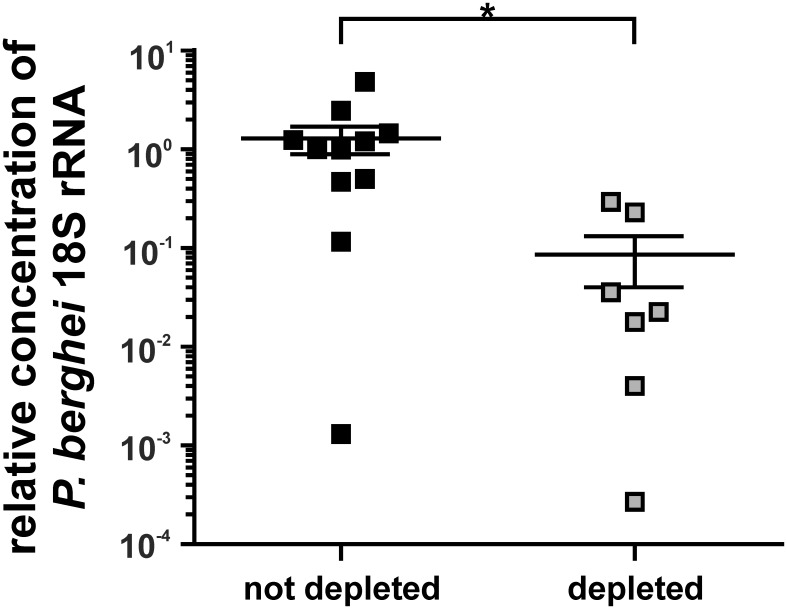
Impact of T_reg_ depletion in parasite burden in the liver. Female Balb/c wild type and DEREG mice were immunized with 20 µg ACT-CSP i.p. on day –7. DT was applied on days –6, −5 and –4; on day 0 mice were infected with 1000 *P. berghei* sporozoites i.v. The *P. berghei* parasite load in the livers was determined 30 h after infection by qPCR. The expression of *P. berghei* 18S rRNA relative to mouse GAPDH expression is shown as mean +/− SEM of n = 11 wild type mice and n = 7 DEREG mice. Statistical significance was analyzed using the Mann–Whitney test.

Due to the very high number of merozoites released by a single infected hepatocyte, a direct correlation between liver-stage burden and parasitemia is unlikely. In order to directly compare the protection induced by vaccination of T_reg_ depleted and not depleted mice, parasite burden in the liver after challenge with *P. berghei* sporozoites was determined using a quantitative PCR (qPCR) specific for *P. berghei* 18S rRNA normalized to a murine housekeeping gene [19]. To this end, DEREG mice and their non-transgenic littermates mice were immunized once with ACT-CSP on day –7. DT was applied on days −6, −5 and −4. On day 0 mice were infected with 1000 *Pb*A sporozoites i.v. In order to prevent contamination of liver tissue with blood-derived *P. berghei*, analysis was performed 30 h post-challenge before the blood stage was established. Despite the limited time for effector functions CSP-specific CD8^+^ T cells to counteract infection, parasite burden in the liver was reduced in Treg depleted mice compared with not depleted mice (Fig. 5).

### Effect of T_reg_ depletion during boost immunization

**Figure 6:**
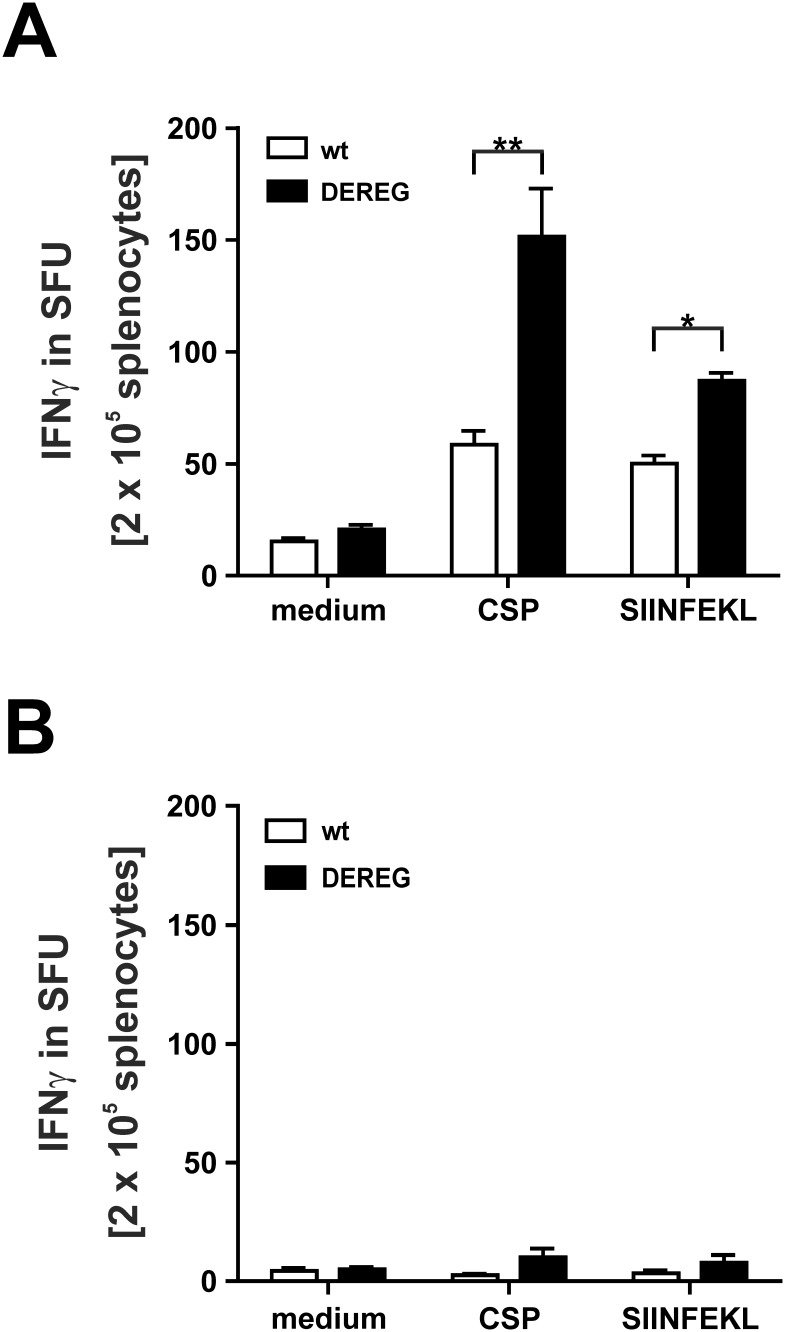
Effect of T_reg_-depletion on the efficacy of a boost immunization. (A) To analyze the effect of T_reg_ depletion on the efficacy of a boost immunization, we vaccinated F1 DEREG mice or their non-transgenic littermates with ACT-CSP on day 0 and day 14. Both groups of mice were treated on days 15, 16 and 17 with DT. On day +25, spleen cells were restimulated with CSP-peptide or OVA-peptide and IFNγ production in spot forming units (SFU) was analyzed using ELISPOT. Data are expressed as mean +**/−** SD of 5 mice. This experiment was repeated twice with similar results. *, p<0.05, p<0.01. (B) A similar experiment was performed using as well F1 DEREG mice and F1 non-transgenic littermates that were treated on days 15, 16 and 17with DT but without previous immunization.

To analyze the effect of T_reg_ depletion on the efficacy of a boost immunization, we vaccinated F1 DEREG mice or their non-transgenic littermates with ACT-CSP on day 0 and day 14. Both groups of mice were treated on days 15, 16 and 17 with DT. As expected we observed a depletion of eGFP^+^ T_reg_ in F1 DEREG mice but not in non-transgenic littermates (data not shown). Again we observed a CSP-specific IFNγ response in both groups of mice, although this response was significantly higher in T_reg_-depleted mice (Fig. 6). This indicates that a transient depletion of T_reg_ increases the number of specific T cells during prime immunization (Fig. 3A) or boost immunization (Fig. 6).

**Figure 7:**
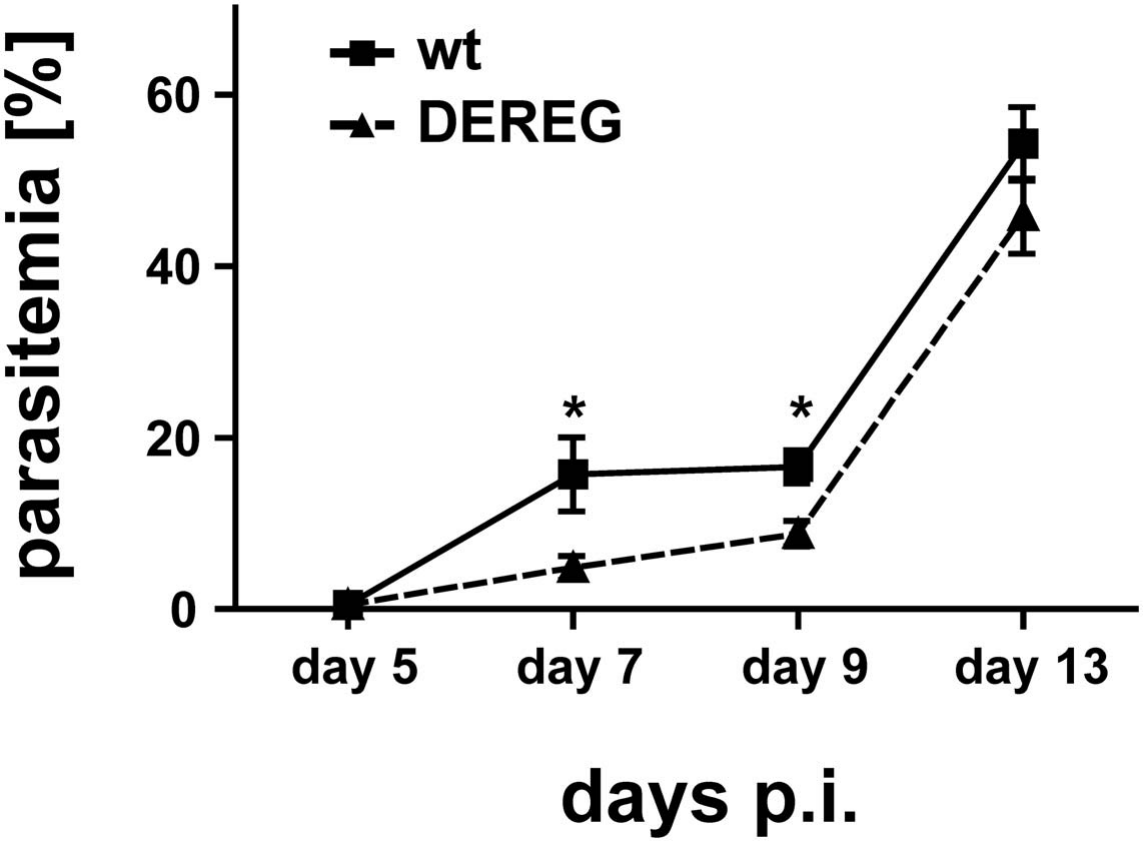
T_reg_-depletion during boost immunization leads to decreased parasitic burden after challenge with *P. berghei* sporozoites. BALB/c DEREG mice or non-transgenic littermates were immunized with ACT-CSP on day 0 and day 14. Both groups of mice were treated on days 15, 16 and 17 with DT. At day +21 mice were challenged i.v. with 1000 *P. berghei* sporozoites and the resulting parasitemia was analyzed at the indicated time points. Data are expressed as mean +**/−** SD of 5 mice per group. This experiment was repeated twice with similar results. *, p<0.05.

In order to investigate if T_reg_ depletion during a boost immunization with ACT-CSP confers protection, we vaccinated BALB/c DEREG mice or non-transgenic littermates with ACT-CSP on day 0 and day 14. Both groups of mice were treated on days 15, 16 and 17 with DT to deplete T_reg_ in DEREG mice. Seven days after the boost immunization mice were challenged with *P. berghei* sporozoites and the resulting parasitemia was analyzed at the indicated time points. A decrease of parasitemia in vaccine-boosted DEREG mice was observed in the course of blood parasitemia on days 7 and 9 after sporozoite challenge (Fig. 7).

### Effect of T_reg_ depletion on CSP-specific memory responses

**Figure 8:**
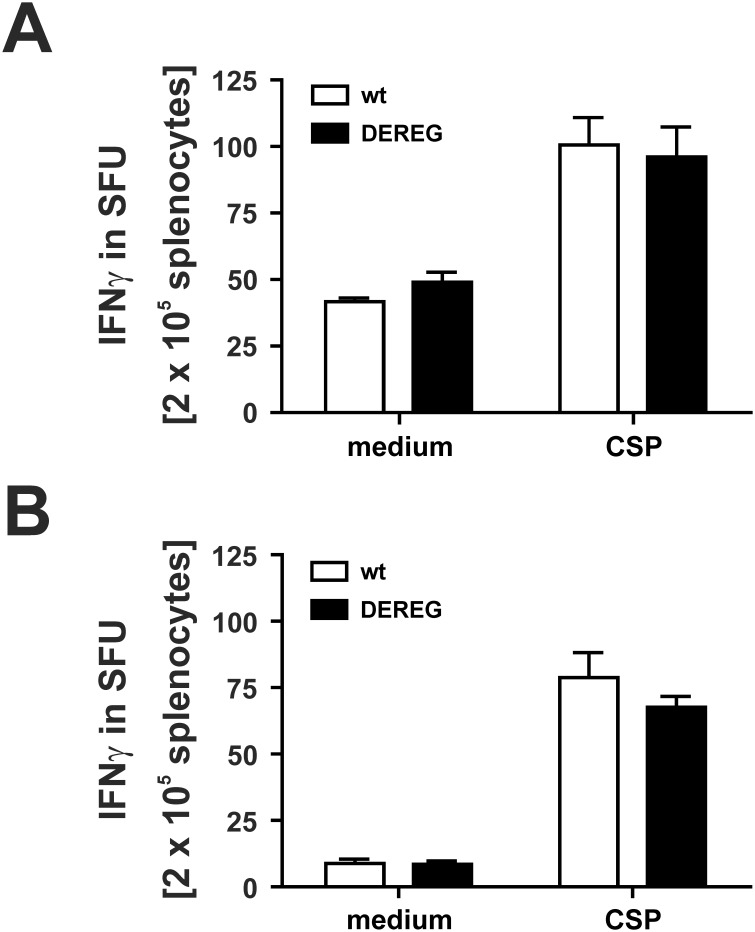
T_reg_-depletion during boost immunization does not affectmemory responses. BALB/c DEREG mice or non-transgenic littermates were immunized with ACT-CSP on day 0 and day 14. Both groups of mice were treated on days 15, 16 and 17 with DT. Spleen cells of mice were restimulated with CSP-peptide after (A) 6 weeks, and (B) 9 weeks. The number of IFNγ producing T cells in spot forming units (SFU) was analyzed by ELISPOT. Data are expressed as mean +**/−**SD of 5 mice per group and time-point. This experiment was repeated once with similar results.

To test whether an enhanced T cell response induced by T_reg_-depletion during immunization is reflected by an increased memory response at later time points after immunization, we analyzed the CSP-specific CD8^+^ T cell response in the spleen 6 weeks and 9 weeks after immunization of BALB/c DEREG-mice, respectively (Fig. 8 A and B). Whereas after immunization the T_reg_-depleted mice exhibit a significantly increased number of CSP-specific cells (Fig. 3), this difference vanished at later time points and no differences between groups were observed anymore at 6 weeks and 9 weeks after immunization. At these time points, T_reg_-depleted and control mice have similar numbers of CSP-specific T cells. This indicates that T_reg_-depletion is accompanied by an increased expansion of antigen-specific T cells but this cell population contracts at the same rate as in control mice.

## Discussion

Recent studies suggest that regulatory T cells (T_reg_) play a central role in the control of the magnitude of an immune response [20]. The modulation of T_reg_ function is an attractive target to increase the efficacy of vaccines but a prerequisite for clinical application is the minimization of possible side effects by designing interventions as specific as possible [21].

Foxp3^+^ T_reg_ express CD25 constitutively and it was shown that anti-CD25 treatment deplete T_reg_ [22], [23]. Several studies used anti-CD25 antibodies to study the function of T_reg_ during infection [15], [24]–[27]. Using this approach it was demonstrated that T_reg_ limit the efficacy of vaccine-induced T cell responses [16], [28], [29]. However, this approach lacks some specificity since not all T_reg_ populations but many non-T_reg_ cell populations express CD25 [20], [30]. Especially during *P. berghei* infection activated CD4^+^ T effector cells are CD25^+^ [18]. Furthermore, the outcome of anti-CD25 depletion is often incomplete and critically depends on the respective tissue and the time-point of antibody application [31]. Using the previously described DEREG mouse it is possible to deplete Foxp3^+^ T_reg_ at selected time points by DT injection [17]. Upon injection on three consecutive days the vast majority of T_reg_ cells were depleted although they started to reappear soon after stopping DT injection. Using this scheme we generated a time window of 3 to 5 days were the numbers of T_reg_ were significantly reduced without observing overt pathology. Foxp3^+^ T_reg_ rapidly reappear after depletion and these reappearing T_reg_ have a similar suppressive potential as the initial T_reg_ population. Thus, DEREG-mice are an ideal model to study the influence of T_reg_ on T cell priming during initial antigen encounter elicited by vaccines, whereas all subsequent steps will take place in the presence of functional T_reg_. In contrast, data on anti-CD25 treatment suggest a long lasting effect of at least 13 to 20 days and therefore not only priming but also expansion and contraction will be influenced by the lack of T_reg_ [31].

We used in the present study ACT-CSP that induces IFNγ producing CD8^+^ T cells against a single epitope of CSP of *Pb* [13]. Although CSP induces a good immune response and confers protection, is also known that in certain models it has a minimal role in sterile protection, because other antigens are responsible for the induction of sterile immunity [32], [33].

The number and function of these T cells can be analyzed by MHC-class I multimer technology and ELISPOT, respectively. Upon depletion of T_reg_ during priming we found a strong enhanced number of these CSP-specific T cells producing IFNγ. It is noteworthy that at the time point of *ex* ****** *vivo* analysis, the T_reg_ population already had reached its original size and was proven to be functional. This suggests that the presence of T_reg_ during initial antigen-encounter limits the number of responding T cells.

Altough T_reg_ depletion exhibited a strong effect on the vaccine-induced response upon primary vaccination and boost immunization, the secondary vaccination did not shown an additional increase in the magnitude of CSP-specific T-cell expansion. The enhanced number of antigen specific CD8^+^ T cells after T_reg_ depletion was only transient, which indicates that the strong induction of T cells in the absence of T_reg_ is followed by a strong contraction of the T cell pool in the presence of T_reg_. Ultimately no differences in the number of CSP-specific T cells were found in both groups several weeks after immunization, although in both groups a significant number of CSP-specific T cells remained detectable. A transient depletion of T_reg_ during boost immunization was also accompanied by an increased number of antigen-specific T cells. However, very similar to the situation observed during priming the differences waned when analyzed at later time points. This result shows that a simple correlation between the number of antigen-specific T effector that were primed initially during immunization and the number of remaining memory T cells does not exist. Hence our data suggest that the efficacy of vaccines, which are intended to induce memory responses, cannot be estimated by analyzing the number of T cells that were primed initially.

T_reg_ were shown to directly interact with DC and to mitigate their maturation process [34], [35]. Previously it was shown that depletion of T_reg_ in DEREG-mice is accompanied by an increase of DC with higher costimulatory activity [36]. Thus T_reg_ might limit the signal strength by preventing maturation of DC, which subsequently influence the priming of T cells. In this scenario a depletion of T_reg_ would augment priming of T cells leading to an increase of T effector cells but a decrease of memory T cells. However, T_reg_ were also shown to interact with T cells by either contact-dependent [37], [38] or independent mechanisms [39]–[41]. Hereby they might influence the expansion of T cells. We observed no increased induction of memory T cells upon depletion of T_reg_. If T effector cells and memory T cells develop in parallel our data would imply that only the size of the T effector cell pool but not the size of the memory T cell pool is regulated by T_reg_. Further studies are needed to pinpoint the exact function of T_reg_ during priming and expansion on the subsequent development of T cell subsets. Whatever the exact function might be, our data have important implications for vaccine development since they indicate that T_reg_ depletion, although leading to an enhanced number of short-lived T effector cells, might not be associated with an enhanced number of long-living T memory cells, which is the major goal of prophylactic vaccines. Hence, transient inhibition of T_reg_ function might be more beneficial when a strong but short-lived immune response is desired for example in therapeutical vaccinations e.g. against tumours where an immune response against self-antigens should be elicited [42].

The infection with *P. berghei* sporozoites is quite a challenge for the induced immune response. Only very few hepatocytes are infected and within 48 h a single infected cell may release up to 30,000 merozoites which is sufficient to trigger the blood-stage of malaria. Thus CD8^+^ T cells specific for antigen presented on MHC-I of hepatocytes must find very few infected cells within a large excess of uninfected cells and combat them in a very narrow time-window. This was demonstrated in a study where different amounts of CSP-specific cells were transferred [43]. Using this approach a threshold of protection was defined. For sterile protection against the liver-stage almost 100 to 1000-fold more T cells were needed in comparison with bacterial or viral infections of the liver. Therefore the requirements on a protective immune response in the *P. berghei* model are very high and probably higher in comparison to human malaria since here the liver-stage lasts much longer [44]. Nevertheless we observed a reduced parasitemia when mice were immunized in the absence of T_reg_. Using qPCR to detect *P. berghei* 18S RNA in the liver 30****h postchallenge, we found that depletion of T_reg_ during immunization was also accompanied by a significant reduction of parasite burden, which indicates that the increased number of CSP-specific CD8^+^ T cells are capable to combat infected hepatocytes. However, the difference in the parasitemia between immunized mice that were depleted for T_reg_ or immunized control mice vanishes when mice were challenged at later time-points (data not shown). This corroborated our observation that the enhanced induction of antigen-specific T cells is not accompanied by an increased level of long-living T cells of a memory subtype.

Mice were challenged at day 7 post-priming or boosting and 3 days after DT injection, a time at which innate immune responses might still interfere with the infection. Since T_reg_ are known to suppress innate immunity [45], it is not certain whether the slight decrease of parasitemia in DEREG mice was due to increased numbers of CD8^+^ T cells and the subsequent decrease in liver-stage burden or due to a more active innate immune response against the blood-stage. Our data cannot support the notion that the reduced parasitemia was mediated by CD8^+^ T cells, because the numbers of IFNγ-producing CD8^+^ T cells at day 7 post primary vaccination (Fig. 3) and at day 7 post secondary vaccination (Fig. 5) were similar. To clarify this, it is important for further investigations to include a control group with mice depleted of CD8^+^ T cells before parasite challenge.

Collectively our results demonstrate that a specific and transient depletion of T_reg_ could enhance a vaccine induced CD8^+^ T cell response during prime and boost immunization. Importantly, the enhanced expansion of T effector cells after T_reg_-depletion was not accompanied by an increased long-lasting memory response, demonstrating that the design of novel vaccine strategies must not only be focussed on the optimisation of a strong short term immune response since this is not necessarily leading to a strong memory response.

## Supporting Information

Figure S1 **Kinetics of T_reg_ depletion in DEREG mice.** The consecutive application of DT i.p. in DEREG mice over three days leads to a depletion of CD4^+^Foxp3^+^ T_reg_ within 72****h. Mice were treated with DT on days +1, +2 and +3. The percentage of CD4^+^Foxp3^+^ T_reg_ in the blood was measured in three F1 DEREG C57BL/6×BALB/c mice by flow cytometry at the indicated time points. Data is expressed as mean +/− SEM in relation to all cells in the lymphocyte gate as defined by the respective forward- and sideward scatter.
(TIF)

## References

[1] Good MF, Doolan DL (1999) Immune effector mechanisms in malaria. Current opinion in immunology 11: 412–419. 10448141 10.1016/S0952-7915(99)80069-7

[2] Balam S, Romero JF, Bongfen SE, Guillaume P, Corradin G (2012) CSP–a model for in vivo presentation of Plasmodium berghei sporozoite antigens by hepatocytes. PloS one 7: e51875. 23272182 10.1371/journal.pone.0051875 PMC3525584

[3] Nussenzweig RS, Vanderberg J, Most H, Orton C (1967) Protective immunity produced by the injection of x-irradiated sporozoites of plasmodium berghei. Nature 216: 160–162. 6057225 10.1038/216160a0

[4] Schofield L, Villaquiran J, Ferreira A, Schellekens H, Nussenzweig R, et al (1987) Gamma interferon, CD8+ T cells and antibodies required for immunity to malaria sporozoites. Nature 330: 664–666. 3120015 10.1038/330664a0

[5] Krzych U, Dalai S, Zarling S, Pichugin A (2012) Memory CD8 T cells specific for plasmodia liver-stage antigens maintain protracted protection against malaria. Front Immunol 3: 370. 23233854 10.3389/fimmu.2012.00370 PMC3517952

[6] Jobe O, Lumsden J, Mueller AK, Williams J, Silva-Rivera H, et al (2007) Genetically attenuated Plasmodium berghei liver stages induce sterile protracted protection that is mediated by major histocompatibility complex Class I-dependent interferon-gamma-producing CD8+ T cells. The Journal of infectious diseases 196: 599–607. 17624847 10.1086/519743 PMC3594113

[7] Trimnell A, Takagi A, Gupta M, Richie TL, Kappe SH, et al (2009) Genetically attenuated parasite vaccines induce contact-dependent CD8+ T cell killing of Plasmodium yoelii liver stage-infected hepatocytes. Journal of immunology 183: 5870–5878. 10.4049/jimmunol.0900302 19812194

[8] Plebanski M, Gilbert SC, Schneider J, Hannan CM, Layton G, et al (1998) Protection from Plasmodium berghei infection by priming and boosting T cells to a single class I-restricted epitope with recombinant carriers suitable for human use. European journal of immunology 28: 4345–4355. 9862371 10.1002/(SICI)1521-4141(199812)28:12<4345::AID-IMMU4345>3.0.CO;2-P

[9] Tartz S, Kamanova J, Simsova M, Sebo P, Bolte S, et al (2006) Immunization with a circumsporozoite epitope fused to Bordetella pertussis adenylate cyclase in conjunction with cytotoxic T-lymphocyte-associated antigen 4 blockade confers protection against Plasmodium berghei liver-stage malaria. Infection and immunity 74: 2277–2285. 16552058 10.1128/IAI.74.4.2277-2285.2006 PMC1418933

[10] Allsopp CE, Plebanski M, Gilbert S, Sinden RE, Harris S, et al (1996) Comparison of numerous delivery systems for the induction of cytotoxic T lymphocytes by immunization. European journal of immunology 26: 1951–1959. 8765044 10.1002/eji.1830260841

[11] Schneider J, Gilbert SC, Hannan CM, Degano P, Prieur E, et al (1999) Induction of CD8+ T cells using heterologous prime-boost immunisation strategies. Immunological reviews 170: 29–38. 10566139 10.1111/j.1600-065x.1999.tb01326.x

[12] Simsova M, Sebo P, Leclerc C (2004) The adenylate cyclase toxin from Bordetella pertussis–a novel promising vehicle for antigen delivery to dendritic cells. International journal of medical microbiology: IJMM 293: 571–576. 15149033 10.1078/1438-4221-00291

[13] Tartz S, Russmann H, Kamanova J, Sebo P, Sturm A, et al (2008) Complete protection against P. berghei malaria upon heterologous prime/boost immunization against circumsporozoite protein employing Salmonella type III secretion system and Bordetella adenylate cyclase toxoid. Vaccine 26: 5935–5943. 18804138 10.1016/j.vaccine.2008.08.057

[14] Nie CQ, Bernard NJ, Schofield L, Hansen DS (2007) CD4+ CD25+ regulatory T cells suppress CD4+ T-cell function and inhibit the development of Plasmodium berghei-specific TH1 responses involved in cerebral malaria pathogenesis. Infection and immunity 75: 2275–2282. 17325053 10.1128/IAI.01783-06 PMC1865737

[15] Haque A, Best SE, Amante FH, Mustafah S, Desbarrieres L, et al (2010) CD4+ natural regulatory T cells prevent experimental cerebral malaria via CTLA-4 when expanded in vivo. PLoS pathogens 6: e1001221. 21170302 10.1371/journal.ppat.1001221 PMC3000360

[16] Moore AC, Gallimore A, Draper SJ, Watkins KR, Gilbert SC, et al (2005) Anti-CD25 antibody enhancement of vaccine-induced immunogenicity: increased durable cellular immunity with reduced immunodominance. Journal of immunology 175: 7264–7273. 10.4049/jimmunol.175.11.7264 16301631

[17] Lahl K, Sparwasser T (2011) In vivo depletion of FoxP3+ Tregs using the DEREG mouse model. Methods in molecular biology 707: 157–172. 21287334 10.1007/978-1-61737-979-6_10

[18] Steeg C, Adler G, Sparwasser T, Fleischer B, Jacobs T (2009) Limited role of CD4+Foxp3+ regulatory T cells in the control of experimental cerebral malaria. Journal of immunology 183: 7014–7022. 10.4049/jimmunol.0901422 19890049

[19] Witney AA, Doolan DL, Anthony RM, Weiss WR, Hoffman SL, et al (2001) Determining liver stage parasite burden by real time quantitative PCR as a method for evaluating pre-erythrocytic malaria vaccine efficacy. Molecular and Biochemical Parasitology 118: 233–245. 11738713 10.1016/s0166-6851(01)00372-3

[20] Sakaguchi S (2011) Regulatory T cells: history and perspective. Methods in molecular biology 707: 3–17. 21287325 10.1007/978-1-61737-979-6_1

[21] Hansen W, Westendorf AM, Buer J (2008) Regulatory T cells as targets for immunotherapy of autoimmunity and inflammation. Inflammation & allergy drug targets 7: 217–223. 19075787 10.2174/187152808786848360

[22] Miyara M, Gorochov G, Ehrenstein M, Musset L, Sakaguchi S, et al (2011) Human FoxP3+ regulatory T cells in systemic autoimmune diseases. Autoimmunity reviews 10: 744–755. 21621000 10.1016/j.autrev.2011.05.004

[23] Rech AJ, Vonderheide RH (2009) Clinical use of anti-CD25 antibody daclizumab to enhance immune responses to tumor antigen vaccination by targeting regulatory T cells. Annals of the New York Academy of Sciences 1174: 99–106. 19769742 10.1111/j.1749-6632.2009.04939.x

[24] Rouse BT, Sarangi PP, Suvas S (2006) Regulatory T cells in virus infections. Immunological reviews 212: 272–286. 16903920 10.1111/j.0105-2896.2006.00412.x

[25] Rad R, Brenner L, Bauer S, Schwendy S, Layland L, et al (2006) CD25+/Foxp3+ T cells regulate gastric inflammation and Helicobacter pylori colonization in vivo. Gastroenterology 131: 525–537. 16890606 10.1053/j.gastro.2006.05.001

[26] Taylor MD, van der Werf N, Harris A, Graham AL, Bain O, et al (2009) Early recruitment of natural CD4+ Foxp3+ Treg cells by infective larvae determines the outcome of filarial infection. European journal of immunology 39: 192–206. 19089814 10.1002/eji.200838727

[27] D’Elia R, Behnke JM, Bradley JE, Else KJ (2009) Regulatory T cells: a role in the control of helminth-driven intestinal pathology and worm survival. Journal of immunology 182: 2340–2348. 10.4049/jimmunol.0802767 PMC2649429 19201888

[28] Furuichi Y, Tokuyama H, Ueha S, Kurachi M, Moriyasu F, et al (2005) Depletion of CD25+CD4+T cells (Tregs) enhances the HBV-specific CD8+ T cell response primed by DNA immunization. World journal of gastroenterology: WJG 11: 3772–3777. 15968737 10.3748/wjg.v11.i24.3772 PMC4316033

[29] Chuang C-M, Hoory T, Monie A, Wu A, Wang M-C, et al (2009) Enhancing therapeutic HPV DNA vaccine potency through depletion of CD4+CD25+ T regulatory cells. Vaccine 27: 684–689. 19056449 10.1016/j.vaccine.2008.11.042 PMC2658755

[30] Couper KN, Lanthier PA, Perona-Wright G, Kummer LW, Chen W, et al (2009) Anti-CD25 antibody-mediated depletion of effector T cell populations enhances susceptibility of mice to acute but not chronic Toxoplasma gondii infection. Journal of immunology 182: 3985–3994. 10.4049/jimmunol.0803053 PMC3942880 19299696

[31] Couper KN, Blount DG, de Souza JB, Suffia I, Belkaid Y, et al (2007) Incomplete depletion and rapid regeneration of Foxp3+ regulatory T cells following anti-CD25 treatment in malaria-infected mice. Journal of immunology 178: 4136–4146. 10.4049/jimmunol.178.7.4136 PMC2235934 17371969

[32] Mauduit M, Tewari R, Depinay N, Kayibanda M, Lallemand E, et al (2010) Minimal role for the circumsporozoite protein in the induction of sterile immunity by vaccination with live rodent malaria sporozoites. Infection and immunity 78: 2182–2188. 20194600 10.1128/IAI.01415-09 PMC2863544

[33] Gruner AC, Mauduit M, Tewari R, Romero JF, Depinay N, et al (2007) Sterile protection against malaria is independent of immune responses to the circumsporozoite protein. PloS one 2: e1371. 18159254 10.1371/journal.pone.0001371 PMC2147056

[34] Mahnke K, Ring S, Bedke T, Karakhanova S, Enk AH (2008) Interaction of regulatory T cells with antigen-presenting cells in health and disease. Chemical immunology and allergy 94: 29–39. 18802334 10.1159/000154854

[35] Tang Q, Krummel MF (2006) Imaging the function of regulatory T cells in vivo. Current opinion in immunology 18: 496–502. 16765579 10.1016/j.coi.2006.05.007

[36] Schildknecht A, Brauer S, Brenner C, Lahl K, Schild H, et al (2010) FoxP3+ regulatory T cells essentially contribute to peripheral CD8+ T-cell tolerance induced by steady-state dendritic cells. Proceedings of the National Academy of Sciences of the United States of America 107: 199–203. 20018763 10.1073/pnas.0910620107 PMC2806715

[37] Piccirillo CA, d’Hennezel E, Sgouroudis E, Yurchenko E (2008) CD4+Foxp3+ regulatory T cells in the control of autoimmunity: in vivo veritas. Current opinion in immunology 20: 655–662. 18926906 10.1016/j.coi.2008.09.006

[38] Nakamura K, Kitani A, Strober W (2001) Cell contact-dependent immunosuppression by CD4(+)CD25(+) regulatory T cells is mediated by cell surface-bound transforming growth factor beta. The Journal of experimental medicine 194: 629–644. 11535631 10.1084/jem.194.5.629 PMC2195935

[39] Kearley J, Robinson DS, Lloyd CM (2008) CD4+CD25+ regulatory T cells reverse established allergic airway inflammation and prevent airway remodeling. The Journal of allergy and clinical immunology 122: 617–624 e616. 18672278 10.1016/j.jaci.2008.05.048 PMC3389733

[40] Chaturvedi V, Collison LW, Guy CS, Workman CJ, Vignali DAA (2011) Cutting Edge: Human Regulatory T Cells Require IL-35 To Mediate Suppression and Infectious Tolerance. The Journal of Immunology 186: 6661–6666. 21576509 10.4049/jimmunol.1100315 PMC3110563

[41] Collison LW, Pillai MR, Chaturvedi V, Vignali DA (2009) Regulatory T cell suppression is potentiated by target T cells in a cell contact, IL-35- and IL-10-dependent manner. Journal of immunology 182: 6121–6128. 10.4049/jimmunol.0803646 PMC2698997 19414764

[42] Vignali DA (2012) Mechanisms of Treg suppression: still a long way to go. Frontiers in Immunology 3. 10.3389/fimmu.2012.00191 PMC3389608 22783262

[43] Schmidt NW, Podyminogin RL, Butler NS, Badovinac VP, Tucker BJ, et al (2008) Memory CD8 T cell responses exceeding a large but definable threshold provide long-term immunity to malaria. Proceedings of the National Academy of Sciences of the United States of America 105: 14017–14022. 18780790 10.1073/pnas.0805452105 PMC2544571

[44] Butler NS, Schmidt NW, Harty JT (2010) Differential effector pathways regulate memory CD8 T cell immunity against Plasmodium berghei versus P. yoelii sporozoites. Journal of immunology 184: 2528–2538. 10.4049/jimmunol.0903529 PMC2904689 20097864

[45] Shanker A (2010) Adaptive control of innate immunity. Immunology letters 131: 107–112. 20394777 10.1016/j.imlet.2010.04.002 PMC2885571

