# Supplementary figures and images for: Depletion of Regulatory T Cells Augments a Vaccine-Induced T Effector Cell Response against the Liver-Stage of Malaria but Fails to Increase Memory
Source: PLoS One. 2014 Aug 12;9(8):e104627. doi: 10.1371/journal.pone.0104627 (PMC4130546; doi:10.1371/journal.pone.0104627)

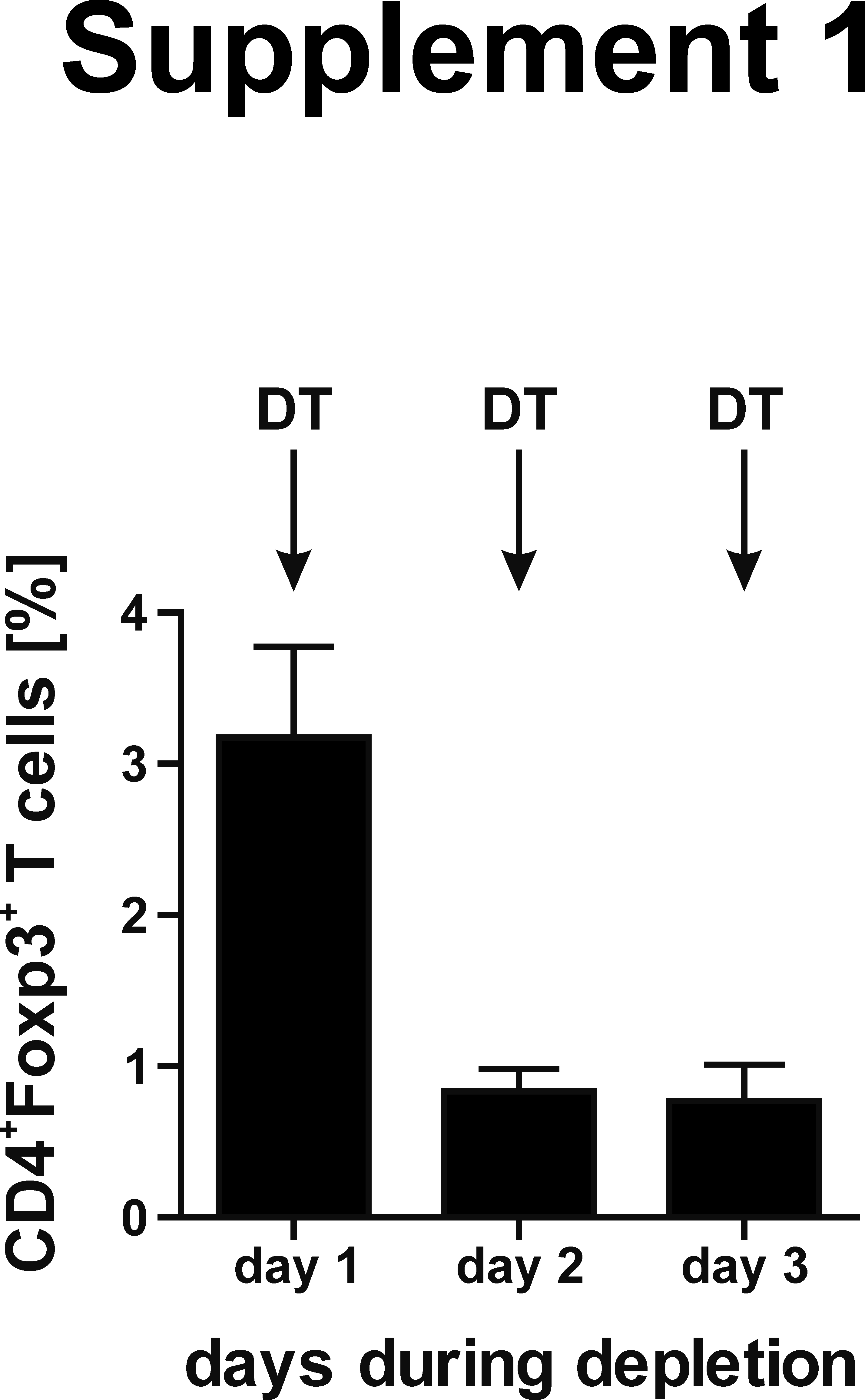

Supplement: Figure S1 — Kinetics of Treg depletion in DEREG mice. The consecutive application of DT i.p. in DEREG mice over three days leads to a depletion of CD4+Foxp3+ Treg within 72h. Mice were treated with DT on days +1, +2 and +3. The percentage of CD4+Foxp3+ Treg in the blood was measured in three F1 DEREG C57BL/6×BALB/c mice by flow cytometry at the indicated time points. Data is expressed as mean +/− SEM in relation to all cells in the lymphocyte gate as defined by the respective forward- and sideward scatter. (TIF) [file pone.0104627.s001.tif]
